# Recovery of Voice After Reconstruction of the Recurrent Laryngeal Nerve and Adjuvant Nimodipine

**DOI:** 10.1007/s00268-017-4235-9

**Published:** 2017-12-28

**Authors:** P. Mattsson, A. Frostell, G. Björck, J. K. E. Persson, R. Hakim, J. Zedenius, M. Svensson

**Affiliations:** 1 Division of Clinical CNS Research, Section of Neurosurgery, Department of Clinical Neuroscience, Karolinska Institutet R2:02, Karolinska University Hospital, 171 76 Stockholm, Sweden; 2 Department of Breast, Endocrine and Sarcoma Tumors, Karolinska University Hospital, 171 76 Stockholm, Sweden; 3 Department of ENT Surgery, Karolinska University Hospital, 171 76 Stockholm, Sweden; 4 Department of Molecular Medicine and Surgery, Karolinska Institutet, Stockholm, Sweden; 5 Department of Neurosurgery, Karolinska University Hospital, 171 76 Stockholm, Sweden

## Abstract

**Background:**

Transection injury to the recurrent laryngeal nerve (RLN) has been associated with permanent vocal fold palsy, and treatment has been limited to voice therapy or local treatment of vocal folds. Microsurgical repair has been reported to induce a better function. The calcium channel antagonist nimodipine improves functional recovery after experimental nerve injury and also after cranial nerve injury in patients. This study aims to present voice outcome in patients who underwent repair of the RLN and received nimodipine during regeneration.

**Methods:**

From 2002–2016, 19 patients were admitted to our center with complete unilateral injury to the RLN and underwent microsurgical repair of the RLN. After nerve repair, patients received nimodipine for 2–3 months. Laryngoscopy was performed repeatedly up to 14 months postoperatively. The Voice Handicap Index (VHI) was administered, and patients’ maximum phonation time (MPT) was recorded during the follow-up.

**Results:**

All patients recovered well after surgery, and nimodipine was well tolerated with no dropouts. None of the patients suffered from atrophy of the vocal fold, and some patients even showed a small ab/adduction of the vocal fold on the repaired side with laryngoscopy. During long-term follow-up (>3 years), VHI and MPT normalized, indicating a nearly complete recovery from unilateral RLN injury.

**Conclusions:**

In this cohort study, we report the results of the first 19 consecutive cases at our center subjected to reconstruction of the RLN and adjuvant nimodipine treatment. The outcome of the current strategy is encouraging and should be considered after iatrogenic RLN transection injuries.

## Introduction

### Recurrent laryngeal nerve transection injury

Transection injury to the recurrent laryngeal nerve (RLN) leads to unilateral vocal cord paralysis (UVCP) with motor, sensory, and autonomic dysfunctions of the larynx such as various degrees of dysphonia, aspiration tendencies, or airway impairment [1, 2].

In the absence of active treatment, patients suffering from RLN transection do not recover voice function [3, 4]. Therefore, a variety of surgical techniques have been used to achieve reinnervation, including RLN-to-RLN anastomosis, ansa cervicalis nerve-to-RLN anastomosis, greater auricular nerve-to-RLN anastomosis, vagal nerve-to-RLN anastomosis, and nerve grafting [3–8]. All of these techniques have been reported to result in an improved voice function on follow-up compared to no treatment, as measured with maximum phonation time (MPT). Even delayed reinnervation (>6 months after the injury) has been reported to improve voice function but with decreasing efficacy with longer intervals between injury and reinnervation [5].

The repair after transection may be associated with a high degree of misguidance, as an additional problem to potential collateral reinnervation from adjacent nerves. Collateral sprouting and misguidance are likely to contribute to poor vocal fold movement [2, 9, 10]. Despite absent ab/adduction after RLN transection, suturing of the nerve seems to result in enhanced recovery of voice [11].

### Adjuvant treatment for peripheral nerve regeneration

Administration of the voltage-gated calcium channel antagonist nimodipine facilitates nerve regeneration and improves functional outcome after peripheral nerve injury [12–18]. The beneficial effect of nimodipine is supported in clinical trials involving cranial nerves [19, 20]. A potential mechanism, suggested by in vivo experiments, is a reduction in the calcium-induced depolarization in the growth cone, which eventually results in extended time for axonal elongation [21, 22]. Administration of nimodipine does improve RLN regeneration and decrease the extent of pathological collateral reinnervation following nerve injuries induced in animal experiments [23]. We previously reported the first human case with a limited, but clear, ab/adduction of the vocal fold after reconstruction of the RLN and administration of nimodipine over three months [24].

### Measurement of voice function

The MPT is the time, in seconds, during which a person can sustain a vowel sound. Normal values for MPT vary greatly between studies. One review of the literature by Maslan et al. (2011) showed mean values between 18 and 30 s for healthy adults (male and female combined) and a range between 6 and 62 s (SD: 5–8 s) [25]. Lacking raw data from these studies, a conservative estimate of the lower normal threshold for MPT could be set at 15 s in the absence of pathology.

The Voice Handicap Index (VHI) was developed by Jacobson et al. (1997) and designed to assess various voice disorders [26]. VHI has been widely utilized in the assessment of vocal fold paralysis [27–30]. The Swedish version of the VHI (Sw-VHI) used in this study was translated and validated by Ohlsson and Dotevall (2009) [31]. The median score was 10 (range 0–38) in normal subjects and 33 (0–80) in patients with voice complaints. The authors suggested 20 points as a cutoff for a pathological score and state that “using 20 points as a cut-off score gives a sensitivity of 0.77 and a specificity of 0.87 for Sw-VHI” [31].

### Aim

In this study, we present aerodynamic and subjective voice outcomes in patients who underwent repair of the RLN and received nimodipine during regeneration.

## Materials and methods

### Patient selection

**Fig. 1:**
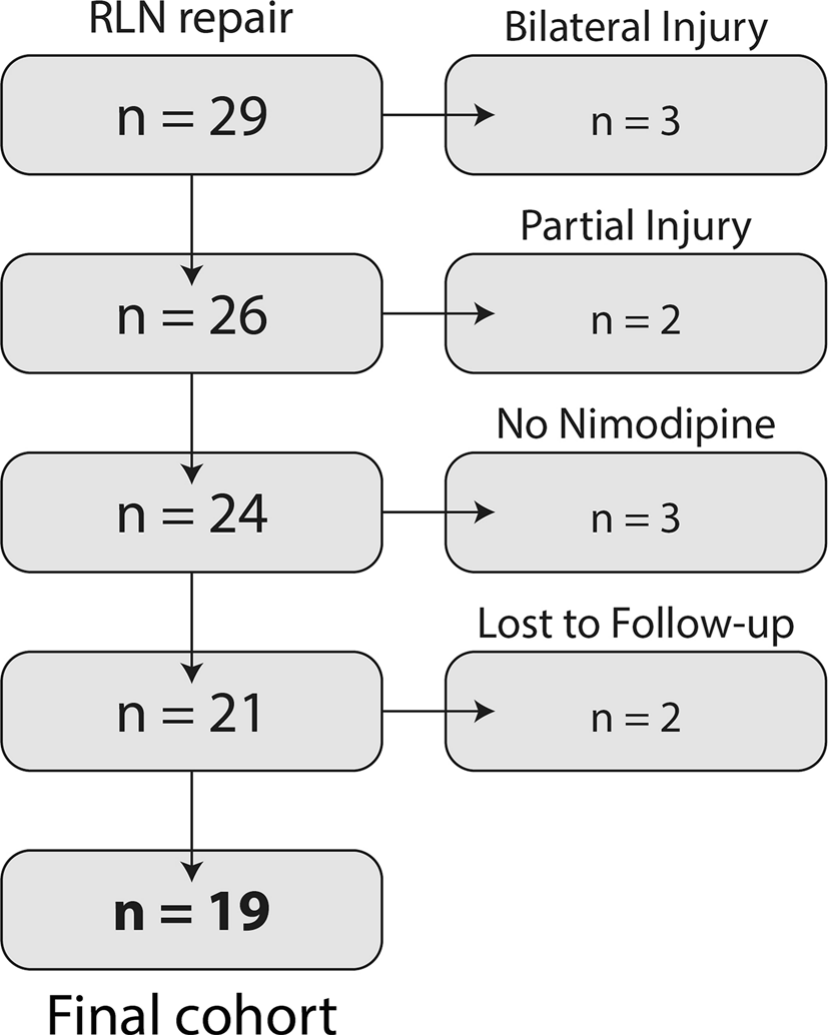
Flowchart describing all patients (*n* = 29) found in the quality register for RLN injury and repair, as well as the reduction of patients to the final cohort (*n* = 19), and the reason for exclusion

We used the local quality register of the Department of Breast and Endocrine Surgery at Karolinska University Hospital (Stockholm, Sweden) to find patients admitted for recurrent laryngeal nerve (RLN) reparation surgery. Of a total of 29 identified patients, 19 contributed data to the study (Fig. 1). Three patients who underwent microsurgical repair but were not treated with nimodipine were excluded from treatment due to: (a) age 10 (deemed too young), (b) repair with direct ansa anastomosis (representing a different neurobiology), and (c) lack of compliance.

Ethical permission was obtained from the local ethics committee for publication of data from the quality register (DNR:2014/596-31/2).

### Microsurgery

All surgical interventions were performed by the same microsurgeon, who is a senior consultant in endocrine surgery and has extensive experience with microsurgical nerve repair from both preclinical and clinical work.

The indication for primary surgery and surgical procedure that gave rise to the RLN injury are presented in Table 1. The injured nerve was identified during the same session or in a new surgical session within 4 days of the first surgery. The timing of surgery varied for practical reasons, and the earliest possible surgery was preferred.

**Table 1:** Characteristics of 19 patients with RLN injury

Age	
Mean (SD)	41.7 (±16.4)
Gender distribution	
Female	14
Male	5
Primary diagnosis	
Goiter	8
Papillary cancer	6
Toxic goiter	2
Parathyroid adenoma	2
Medullary cancer	1
Primary surgery	
Thyroidectomy	11
Hemithyroidectomy	5
Extirpation of parathyroid gland	2
Lymph nodes, reoperation	1
Injury type	
Complete transection	19
Timing of RLN repair	
Same session	10
1 day after injury	5
2 days after injury	2
3 days after injury	1
4 days after injury	1
Type of RLN repair	
Raphy	7
Graft (ansa cervicalis nerve)	7
Graft (sural nerve)	5
Nimodipine treatment time	
Median (range)	3 (2–12)
Speech and language therapy	
No	12
Yes	7

The nerve endings of the proximal and distal stump were trimmed under a microscope (usually 1–2 mm of nerve was removed) to encourage a transverse ending with visually unaffected axonal tissue. The distal stump was trimmed until stimulation with a 1 mA pulse gave a clear EMG signal that was registered from electrodes on an endotracheal tube adjacent to the vocal muscle (intraoperative nerve monitoring, IONM). The distal stump gave a clear signal up to 4 day postoperatively (using IONM in all cases).

When deemed necessary, the gap between proximal and distal ends was filled with an autologous peripheral nerve graft (ansa cervicalis or sural nerve) in order to avoid stretching of the anastomosis. The cervical ansa was chosen when the dimensions corresponded to the size of the RLN. If the ansa cervicalis was too small, the sural nerve was harvested and used instead. Both nerve anastomosis were aligned under a microscope by 2–4 epineural sutures (9–0 Prolene®), enveloped in a layer of hemostatics (Surgicel®), and further armed with fibrin sealant (Tisseel®).

### Postoperative regime and voice follow-up

Patients were prescribed nimodipine (Nimotop®) at 30 mg × 2 three times daily for 2–4 months (Table 1). Flexible laryngoscopy with stroboscopic examination was performed at the first postoperative day and then repeatedly up to 14 months postoperatively. The laryngoscopic examination did not follow a standardized study protocol but was done as clinical routine by a senior laryngoscopist. Vocal fold movement was judged during phonation and inspiration, and ab/adduction was compared to the contralateral side by subjective assessment by the investigator.

### Voice outcome

In this study, for measurements of MPT, the vowel “a” was used at a loudness and pitch that the patient found comfortable. Each patient was given 3–5 attempts, and the longest sustained phonation time was registered [32]. For subjective assessment of voice, we used the VHI translated to Swedish and validated by Ohlsson et al. (2009) [31]. VHI was not measured at baseline. MPT was only recorded for 11 out of 19 patients at baseline.

At the end of the follow-up period, all patients were assessed with MPT and VHI. The longest available follow-up time for VHI and MPT was used in the current study.

### Electrophysiology

From 2–4 weeks postoperatively, most patients (*n* = 14) were examined with a laryngeal electromyogram (EMG) by inserting a thin needle percutaneously into the vocal muscle bilaterally. Information was registered about the innervation to the vocal muscle.

EMG was performed at this point because deinnervation and Wallerian degeneration would be manifest regardless of whether the transected nerve was reconstructed. This time frame was chosen to avoid false negatives from less severe injuries to the RLN or superior laryngeal nerve (e.g., stretching) that might have resulted in a temporary conduction blockade.

### Statistical analysis

Descriptive statistics were presented with mean and standard deviation or median with range, depending on the distribution of the sample. We calculated Spearman’s rank correlation for VHI and MPT vs age at injury and follow-up time. The nonparametric Spearman’s rank correlation was used instead of Pearson’s product-moment correlation because data were sparse and showed both nonlinearity and possible outliers. To visualize trends, we fitted a generalized additive model (GAM) to the data points with the formula y ~ s (x, *k* = 3) through the stat_smooth function of the package ggplot2 in R.

We used a two-tailed paired samples *t* test to compare MPT at baseline with MPT at follow-up in the subsample containing both measurements (*n* = 11). The null hypothesis was no mean difference between these time points.

The statistical significance threshold was set at *p* < 0.05 for all inferential tests. Statistical analyses and visualizations were done in R (version 3.3.3) [33] using packages *ggplot2* [34] *and cowplot* [35].

## Results

### Surgical and medical complications

All 19 patients recovered well after surgery, with no evidence of postoperative infections. All patients in the current series tolerated nimodipine treatment well. Some patients described suspicious orthostatic episodes but continued the treatment. Drugs for hypertension were withdrawn in some patients in order to maintain blood pressure (Table 1).

One patient experienced a small area of permanent loss of sensory function in the lateral foot and intermittent paresthesia in the area after sural nerve grafting. Symptoms were relieved by injection of local anesthetics in the probable location of a possible neuroma. Another patient suffered cramps of the larynx with intermittent subjective breathing difficulties (despite unilateral RLN injury) and poor voice quality despite normal MPT and absence of a glottal gap. Symptoms were relieved with botulinum toxin injections in the vocal folds and anxiolytic medications.

### Electrophysiology

Electrophysiology was performed in 14 out of 19 patients 2–4 weeks postoperatively. The vocal muscle (thyroarytenoid, TA) on the uninjured side showed normal voluntary activation. On the injured side, denervation was detected to varying degrees by the presence of positive sharp waves and fibrillation potentials in combination with absence of voluntary recruitment of motor units. Out of 14 patients, 12 patients (86%) showed a denervation pattern with positive sharp waves and fibrillations. Surprisingly, two patients (14%) showed no signs of denervation, despite an obvious UVCP detected by intraoperative monitoring, postoperative laryngoscopy, and loss of voice function.

### Flexible laryngoscopy

All patients were examined with flexible laryngoscopy immediately after surgery and intermittently for up to 14 months after surgery.

On day 1 postoperatively, all patients had a breathy voice, various degrees of glottal gap, and an immobile vocal fold on the injured and reconstructed side. At the 12-month follow-up, all patients showed efficient closing and no glottal gap. Some patients (*n* = 8, 42%) showed a small ab/adduction of the injured side during phonation, and 1 of those 8 patients showed nearly normal motility of the vocal fold. None of the patients had an atrophic vocal fold at any point during the follow-up, and none of the patients required further treatment with local injections in the vocal fold.

### Voice outcome

VHI was not measured at baseline, and measurements of MPT were only available for 11 out of 19 patients at baseline. The mean MPT at baseline was 9.0 (±5.2) seconds and 22 (±7.7) seconds at follow-up. A paired samples t test showed a mean difference of 13 s and was significant at *p* < 0.001.

**Fig. 2:**
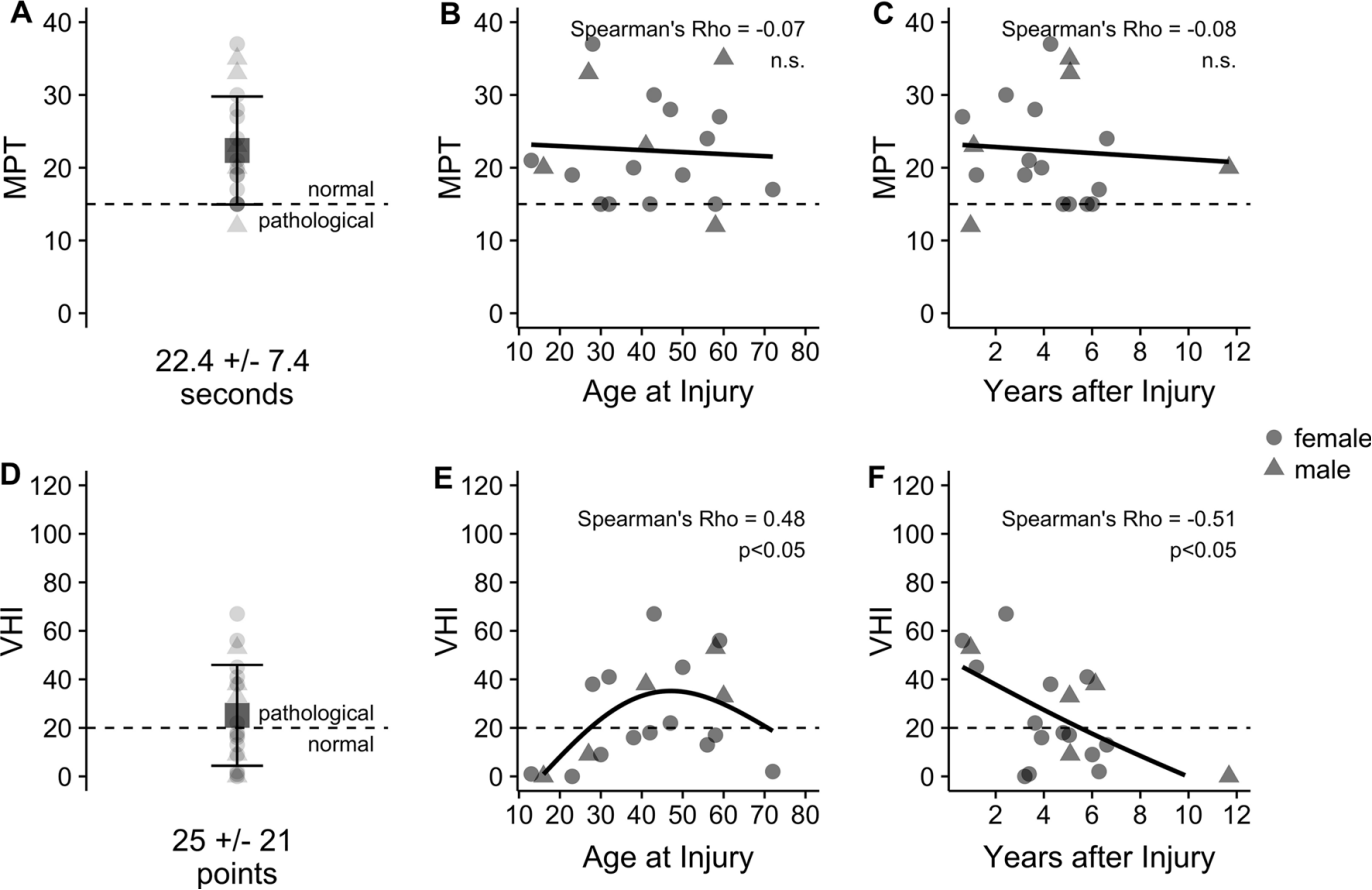
Objective outcome shown by maximum phonation time, MPT (**a**–**c**), and subjective outcome shown by voice handicap index, VHI (**d**–**f**), after RLN repair and adjuvant nimodipine. VHI was positively correlated with age at injury—with older patients describing a poorer subjective voice outcome than younger (**e**)—and negatively correlated to follow-up time—with better subjective voice outcomes in longer follow-up times. MPT was not correlated with VHI, age at injury (**b**), or follow-up time (**c**)

At follow-up, for the whole cohort of 19 patients, the mean MPT was 22.4 (±7.4) seconds (Fig. 2a) after a mean follow-up time of 4.3 (±2.6) years. VHI was 25 (±21) points after a mean follow-up time of 4.5 (±2.5) years (Fig. 2d).

Patients with a follow-up time of more than 3 years (*n* = 14) showed a better subjective outcome with VHI 16 (±14) points, despite a lack of improvement in MPT at 22.4 (±7.8) seconds.

Using Spearman’s rank correlation, VHI was correlated with age at injury (*p* < 0.05, Fig. 2e) and time to follow-up (*p* < 0.05, Fig. 2f) but not with MPT (*p* = 0.68). MPT was not correlated with age (*p* = 0.78, Fig. 2b) or time to follow-up (*p* = 0.76, Fig. 2c).

All values are given as means and standard deviations; see Fig. 2a–e for further details.

## Discussion

The aim of the present study was to present voice outcome in patients who were treated with graft or raphy in combination with adjuvant nimodipine following complete transection of the recurrent laryngeal nerve (RLN).

To our knowledge, this is the first study that presents both objective and subjective outcomes after RLN injury and repair in adult patients as represented by maximum phonation time (MPT) and voice handicap index (VHI). Patients had a near-normal MPT 1 year after injury, and VHI continued to improve and approached normal at >3 years after injury. Since there was no control group, it is unclear whether to what degree the observed improvement over time in our data is a result of the treatment, or a general phenomenon after RLN injury.

Importantly, none of the patients in the cohort suffered from atrophy of the injured vocal fold, and therefore, none of the 19 patients needed further surgical treatment (e.g., injections in the vocal fold).

After transection injury, treatment with RLN repair and adjuvant nimodipine is safe. No severe side effects or complications were reported. Nerves were repaired up to 4 days postinjury, and intraoperative EMG showed the distal stump to be functional in all patients at surgery. Voice outcome was similar despite timing of surgery (data not shown). During follow-up, a majority of the patients described a distinct stepwise improvement in overall voice function instead of a slow, continuous improvement. This supports the theory that reinnervation rather than adaption or other mechanisms is responsible for the improvement.

A small but existing ab/adduction of the vocal fold emerged in several patients (*n* = 8, 42%) on laryngoscopy. All these patients showed a complete denervation pattern on EMG 2–4 weeks after injury. However, some patients had a less complete denervation pattern or even normal EMG results (*n* = 2). Most likely, this finding was represented by partial innervation by the superior laryngeal nerve (SLN), in line with human dissection studies describing dual innervation (RLN and SLN) to the laryngeal muscles [36–39]. A recent study by De Virgilio et al. failed to show any difference in voice outcome for patients with RLN injury with or without concomitant SLN injury, so the clinical importance of dual innervation remains unclear [40].

### Limitations

The current study suffers from several important limitations. First, all patients were given the same treatment. Therefore, the additional effect of nimodipine in RLN injury and repair remains uncertain. A second limitation is the potential lack of compliance since patients were required to take three daily doses of nimodipine for 3 months. Finally, as a retrospective description of patient outcomes, the study involves a heterogeneous follow-up routine and the possibility of bias.

## Conclusion

Acute reinnervation of the RLN with graft or raphy in combination with nimodipine treatment is safe and results in excellent long-term voice outcomes after RLN injury. Patients’ subjective assessment of voice function (VHI) continued to improve up to 3 years posttreatment.

Since this is a retrospective study without control group, a randomized controlled trial would be needed to elucidate fully the effect of surgery and nimodipine.
